# Aidi injection reduces doxorubicin-induced cardiotoxicity by inhibiting carbonyl reductase 1 expression

**DOI:** 10.1080/13880209.2022.2110127

**Published:** 2022-08-18

**Authors:** Yuan Lu, Wen Liu, Ting Lv, Yanli Wang, Ting Liu, Yi Chen, Yang Jin, Jin Huang, Lin Zheng, Yong Huang, Yan He, Yongjun Li

**Affiliations:** a State Key Laboratory of Functions and Applications of Medicinal Plants, Guizhou Provincial Key Laboratory of Pharmaceutics, Guizhou Medical University, Guiyang, China; b The Affiliated Hospital of Guizhou Medical University, Guiyang, China; c Engineering Research Center for the Development and Application of Ethnic Medicine and TCM (Ministry of Education), Guizhou Medical University, Guiyang, China; d School of Pharmacy, Guizhou Medical University, Guiyang, China

**Keywords:** Traditional Chinese medicine, doxorubicinol, H9c2 cells, UPLC-MS/MS

## Abstract

**Context:**

Aidi injection (ADI), a traditional Chinese medicine antitumor injection, is usually combined with doxorubicin (DOX) for the treatment of malignant tumours. The cardiotoxicity of DOX is ameliorated by ADI in the clinic. However, the relevant mechanism is unknown.

**Objective:**

To investigate the effects of ADI on DOX-induced cardiotoxicity and its mechanism.

**Materials and methods:**

ICR mice were randomly divided into six groups: control, ADI-L, ADI-H, DOX, DOX + ADI-L and DOX + ADI-H. DOX (*i.p.*, 0.03 mg/10 g) was administered in the presence or absence of ADI (*i.p.,* 0.1 or 0.2 mL/10 g) for two weeks. Heart pathology and levels of AST, LDH, CK, CK-MB and BNP were assessed. H9c2 cells were treated with DOX in the presence or absence of ADI (1, 4, 10%). Cell viability, caspase-3 activity, nuclear morphology, and CBR1 expression were then evaluated. DOX and doxorubicinol (DOXol) concentrations in heart, liver, kidneys, serum, and cells were analysed by UPLC-MS/MS.

**Results:**

High-dose ADI significantly reduced DOX-induced pathological changes and the levels of AST, LDH, CK, CK-MB and BNP to normal. Combined treatment with ADI (1, 4, 10%) improved the cell viability, and IC50 increased from 68.51 μM (DOX alone) to 83.47, 176.9, and 310.8 μM, reduced caspase-3 activity by 39.17, 43.96, and 61.82%, respectively. High-dose ADI inhibited the expression of CBR1 protein by 32.3%, reduced DOXol levels in heart, serum and H9c2 cells by 59.8, 72.5 and 48.99%, respectively.

**Discussion and Conclusions:**

ADI reduces DOX-induced cardiotoxicity by inhibiting CBR1 expression, which provides a scientific basis for the rational use of ADI.

## Introduction

Aidi injection (Z52020236, ADI), a traditional Chinese medicine injection, was approved by the China Food and Drug Administration (CFDA) in 1996 for the treatment of malignant tumours, including primary liver cancer, lung cancer, rectal cancer, malignant lymphoma and gynecological malignancies. The ingredients of ADI are extracts from *Mylabris phalerata* Pallas (Meloidae), *Astragalus membranaceus* (Fisch.) Bge. (Fabaceae), *Panax ginseng* C. A. Mey. (Araliaceae) and *Acanthopanax senticosus* (Rupr. et Maxim.) Harms (Araliaceae). It has exhibited good efficacy, safety and cost-effectiveness in clinical treatment (Xiao et al. 2016, 2017; Xie et al. 2019), and has been included in the "National Essential Medical Drug List" in China for many years. ADI is usually combined with anthracyclines such as doxorubicin (DOX), epirubicin, or pirarubicin to treat malignant tumours (Wang et al. 2016; Dai et al. 2018; Dou et al. 2020). Clinical studies have found that it can enhance tumour sensitivity to chemotherapy drugs, enhance immunity, reduce toxicity and side effects, and that it exhibits significant synergy (Xiao et al. 2016, 2017; Xie et al. 2019). However, reports of ADI reducing the side effects of chemotherapy drugs are mostly clinical observations, and basic research is lacking. Furthermore, there are no reports on the mechanism by which ADI reduces the cardiotoxicity of anthracyclines.

In recent years, a large number of clinical and experimental studies have shown that many traditional Chinese medicines, such as *P. ginseng* and *A. membranaceus* protect against cardiotoxicity caused by DOX (Yu et al. 2018). Both *P. ginseng* and *A. membranaceus* are components of ADI. Studies have shown that ginsenoside Rg1 may improve DOX-induced cardiac dysfunction by inhibiting endoplasmic reticulum stress and autophagy (Li et al. 2017; Xu et al. 2018). Ginsenoside Rg3 antagonises DOX-induced cardiotoxicity by improving endothelial dysfunction caused by oxidative stress via upregulation of the Nrf2-ARE pathway through activation of Akt (Wang et al. 2015). *Astragalus* polysaccharide suppresses DOX-induced cardiotoxicity by regulating the PI3K/Akt and p38MAPK pathways (Cao et al. 2014). Although the above evidence indicates that the *P. ginseng* and *A. membranaceus* in the ADI formula have the potential to reduce DOX-mediated cardiotoxicity, it is still necessary to prove that ADI is able to reduce DOX-induced cardiotoxicity and determine the mechanism. This will provide scientific evidence to support the clinical combination of ADI and DOX.

## Materials and methods

### Chemicals and reagents

ADI (lot number: 20180522) was supplied by Guizhou Ebay Pharmaceutical Co., Ltd. (Guizhou, China). DOX (purity > 98%) and the internal standard (IS), azithromycin (purity > 98%), were purchased from Dalian Meilun Biotech Co., Ltd. (Liaoning, China). DOXol was obtained from Glpbio (Montclair, CA, USA). Hoechst 33342 was purchased from Beyotime Institute of Biotechnology (Haimen, China). Aspartate aminotransferase (AST), lactate dehydrogenase (LDH), creatinine kinase (CK), CK-MB isoenzyme and caspase-3 activity assay kits were provided by Nanjing Jiancheng Bioengineering Institute (Nanjing, China). A mouse BNP (brain natriuretic peptide) ELISA kit was purchased from Shanghai MLBIO Biotechnology Co., Ltd. (Shanghai, China). BCA assay kit, CBR1 and GAPDH antibody were purchased from Abcam (Cambridge, UK). High performance liquid chromatography (HPLC)-grade acetonitrile, methanol and formic acid were supplied by Merck Company Inc. (Darmstadt, Germany). Distilled water was obtained from Watsons Group Co., Ltd. (Hong Kong, PRC). All other chemicals and reagents used were of chromatographic or analytical grade.

### Animals and ethical approval

All experimental procedures were approved by the Institutional Animal Ethics Committee and the Experiment Animal Centre of Guizhou Medical University (animal ethics approval number: 1801207). The *in vivo* efficacy of ADI was evaluated using male ICR mice (18–22 g) from Chongqing Tengxin Biotechnology Co., Ltd. (Chongqing, China, Animal licence number SCXK (Chongqing) 2012-0008). Each cage contained 10 mice that were acclimatised for one week before the experiment. The animal room was well ventilated with sufficient light, room temperature of 18–25 °C and relative humidity of 50–70%. Feeding and management were carried out in strict accordance with the requirements and rules for treatment of laboratory animals.

### Animal treatment

**Figure 1.**
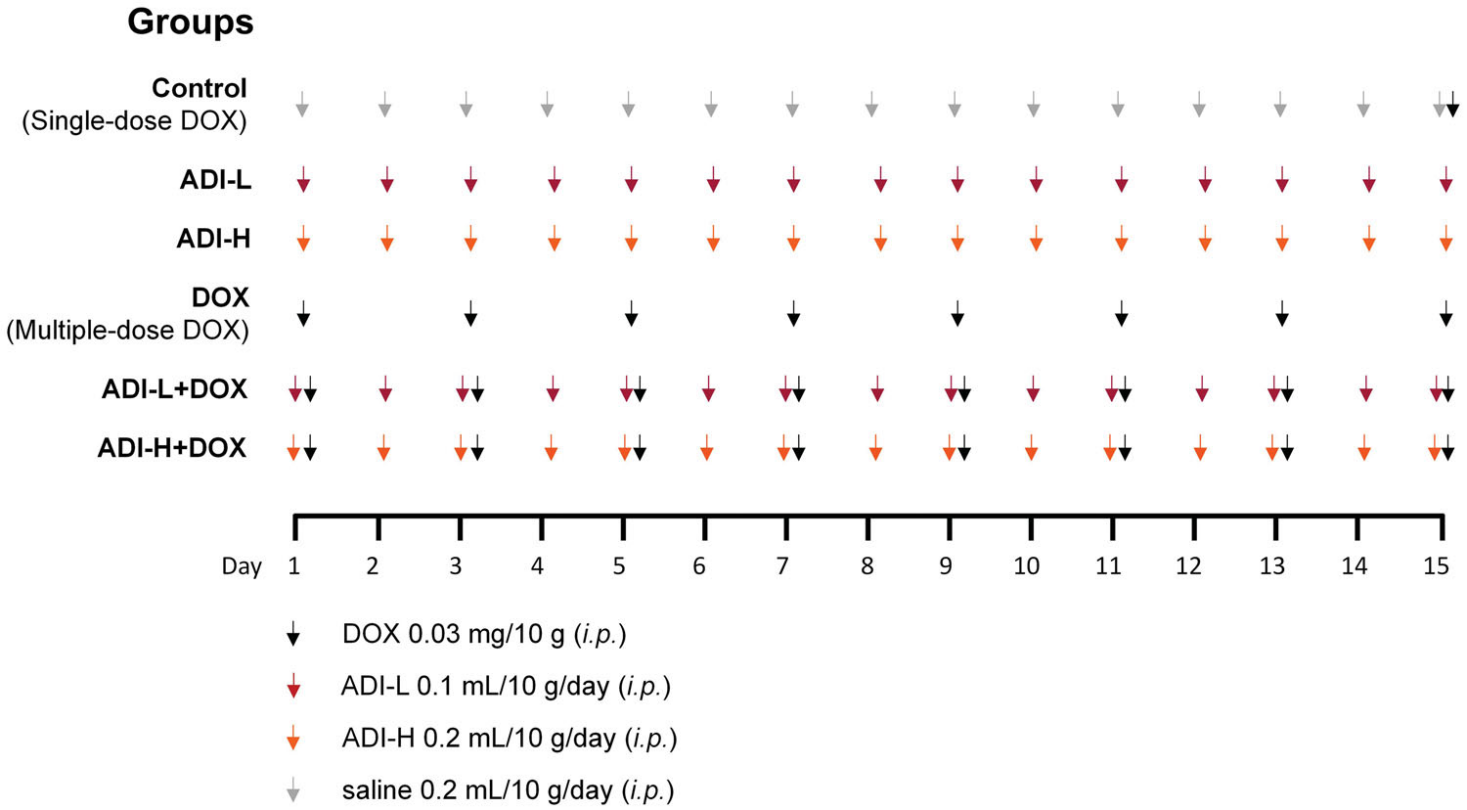
The scheme of experiment grouping and treatments *in vivo.*

Male ICR mice were weighed and randomly divided into six groups (*n* = 6, day 0): (1) saline 0.2 mL/10 g/day (*i.p.*); (2) low-dose ADI (ADI-L, 0.1 mL/10 g/day, *i.p.*); (3) high-dose ADI (ADI-H, 0.2 mL/10 g/day, *i.p.*); (4) DOX 0.03 mg/10 g (*i.p.*, every other day); (5) DOX 0.03 mg/10 g (*i.p.*, every other day) + ADI-L 0.1 mL/10 g/day (*i.p.*); and (6) DOX 0.03 mg/10 g (*i.p.*, every other day) + ADI-H 0.2 mL/10 g/day (*i.p.*). ADI or saline was given 1 h before DOX. Experiment grouping and treatments were performed according to Figure 1. The body weight, feeding behaviour and motor activity of each animal were recorded. The last day, the control group mice were given a single dose of DOX (*i.p.*, 0.03 mg/10 g). All mice were euthanized by isoflurane overdose 2 h after DOX administration. The blood, heart, liver and kidneys were collected and stored at −20 °C for quantification of DOX and DOXol. Specimens from each heart were used for histological examination. Serum samples were used to determine the levels of AST, LDH, CK, CK-MB and BNP.

### Histological examination of the heart

The heart tissues were fixed in 10% formalin, embedded in paraffin and then sections (5 μm thick) were stained with hematoxylin-eosin (H&E). Images of the stained sections were obtained using a light microscope (Nikon Eclipse TE2000-U, Japan) with 100*×* magnification.

### Measurement of AST, LDH, CK, CK-MB and BNP levels

Myocardial cellular damage was evaluated by measuring AST, LDH, CK and CK-MB activity in serum using commercially available assay kits. The serum levels of BNP were determined using the commercial kit, according to the manufacturer’s instructions.

### Quantification of doxorubicin and doxorubicinol by UPLC-MS/MS

Briefly, simple protein precipitation with methanol was performed (Lu et al. 2018). Serum, tissue homogenate or cell lysate (50 µL) was mixed with internal standard solution (20 μL, 1 μg/mL azithromycin). Protein precipitation was initiated by addition of methanol containing 5% formic acid (500 μL). After vortex-mixing for 5 min, samples were centrifuged for 10 min at 18,000*g*. The upper organic phase was transferred into a tube and evaporated to dryness under a gentle stream of nitrogen. The residue was dissolved in the initial mobile phase (500 μL) and centrifuged at 18,000*g* for 10 min. Subsequently, a sample (2 μL) was injected into the UPLC-MS/MS consisting of an Acquity H-Class UPLC system coupled to a Xevo TQ-S Micro Tandem Mass Spectrometer (Waters, Milford, MA, USA). Chromatographic separation was performed on an Acquity UPLC BEH C18 column (2.1 × 50 mm, 1.7 μm particle size, Waters). The column and autosampler tray temperatures were at 45 and 25 °C, respectively. The mobile phase consisted of 0.1% formic acid in acetonitrile (A) and 0.1% formic acid in water (B). A linear gradient at a flow rate of 0.30 mL/min was run at 10–90% A over 0–3.5 min, 90% A over 3.5–4.0 min, 90–10% A over 4.0–4.5 min, and 10% A over 4.5–5.0 min. The MS was operated in positive electrospray ionisation mode. The MS parameters were as follows: source temperature 150 °C, desolvation temperature 500 °C, nitrogen gas flow 1000 L/h and capillary voltage 1.5 kV. Quantification was performed using selective reaction monitoring mode: *m/z* 544.7 → 361.0 (DOX); *m/z* 545.6 → 362.9 (DOXol); *m/z* 749.7 → 591.4 (IS). The cone voltages for DOX, DOXol, and IS were 25, 30, and 30 V, respectively. Collision energies for DOX, DOXol, and IS were all 25 eV.

### Cell culture

Rat H9c2 cells were obtained from the American Type Culture Collection (Rockville, MD, USA) and cultured in Dulbecco’s modified Eagle medium supplemented with 10% foetal bovine serum, 100 IU/mL penicillin and 100 μg/mL streptomycin at 37 °C under 5% CO_2_. Cells were grown in 75 cm^2^ flasks and passaged twice a week at 80% confluency.

### Cell growth inhibition assay

The effects of DOX, DOXol and ADI on cell viability were determined by the MTS assay (Shanghai Promega Biotechnology Co., Ltd.) after incubation of the cells with various concentrations of DOX (1, 5, 10, 20, 50 and 100 μM) in the absence or presence of ADI (1, 4 and 10%) at 37 °C for 24 h. The absorbance was measured at 470 nm and used to calculate the relative ratio of cell viability. The concentrations required to inhibit growth by 50% (IC_50_) were calculated from survival curves.

### Apoptosis assays

Cells were incubated with various concentrations of ADI (1, 4 and 10%) in the absence or presence of DOX (5 μM) at 37 °C for 24 h. Activity of caspase-3, a key enzyme marker of apoptosis, was measured using a commercial assay kit according to the manufacturer's protocol. After DOX (5 μM) and ADI (1, 4 and 10%) treatment, H9c2 cells were incubated with 10 μg/mL Hoechst 33342 for 10 min after fixing with 4% formaldehyde. Nuclear morphology was observed on a Nikon TE2000 microscope (Japan). Representative images were captured by NIS-Elements F (4.60.00 version) software.

### Quantification of DOX and DOXol concentrations in H9c2 cells

H9c2 cells were seeded in 24-well plates. When confluence reached 90%, the cells were incubated with DOX (5 μM) and ADI (1, 4, and 10%) for 1, 2, 4, 8, 12 and 18 h. Subsequently, the medium was removed and cells were washed twice with phosphate-buffered saline. After centrifugation at 500*g* for 5 min (for trypsin removal), cells were suspended in purified water (1 mL) and broken by repeated freeze-thawing. After determination of the protein concentration using a BCA Protein Assay Kit, the concentrations of DOX and DOXol were then determined by UPLC-MS/MS. All experiments were conducted six times.

### Western blot analysis

H9c2 cells were incubated with ADI (10%) or an equivalent volume of PBS for 18 h. Subsequently, protein samples were extracted from cellular lysate. Protein concentration was quantified by BCA assay, then measured by absorption in a spectrophotometer. After that, CBR1 protein was assessed by western blot as described (Endo et al. 2021). Briefly, the protein was separated by SDS-polyacrylamide gel electrophoresis and transferred to a polyvinylidene fluoride transfer membrane (PVDF, Millipore, Bedford, MA, USA). The membranes were blocked with 5% skimmed milk for 1 h at room temperature and then incubated with the corresponding primary antibodies against CBR1 (1:1000) and GADPH (1:5000) overnight at 4 °C. The membrane was then incubated with horseradish peroxidase-conjugated secondary antibody for 2 h at room temperature. After washing with TBST 3 times (5 min each), the membranes were photographed using a gel imager (Thermo Fisher Scientific). All the target proteins were normalised by GADPH and calculated as the percentage of the control.

### Data analysis

Data are expressed as the mean ± standard deviation. Statistical analysis included the two-tailed Student’s *t*-test and one-way ANOVA. Significance was accepted when the *P*-value was less than 0.05 (*P < 0.05; **P < 0.01; ***P < 0.001).

## Results

### ADI reduces doxorubicin-induced myocardial toxicity in mice

**Figure 2.**
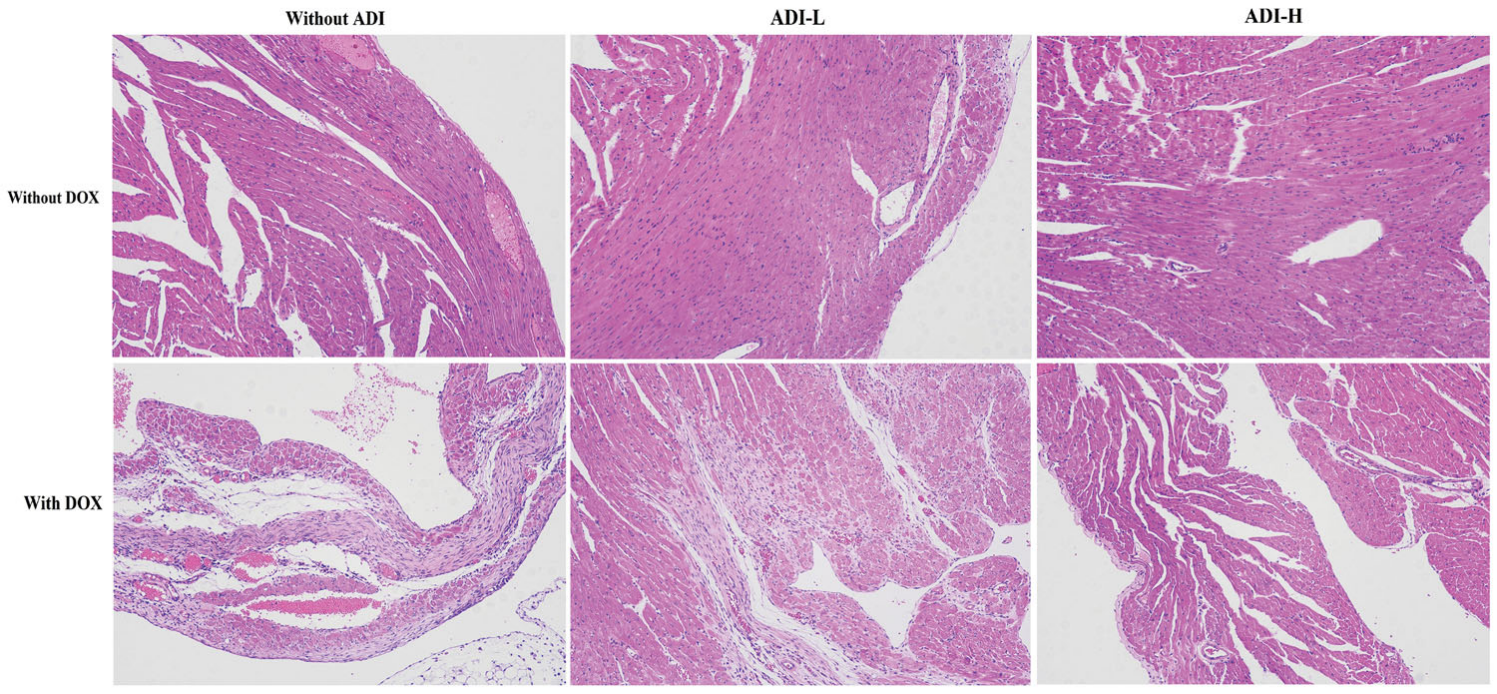
H&E staining images of heart tissues in mice treated with DOX (0.03 mg/10 g) and/or ADI (0.1 and 0.2 mL/10 g) (100*×* magnification). Cells in the control group were in good condition and neatly arranged. In the DOX treatment group, myocardial cell disorders and increased cellular gaps were observed. In the ADI treatment group, no significant changes in cell morphology were seen. In the DOX combined with ADI group, injuries to the cells were slightly reduced.

As shown in Figure 2, heart tissue myocardial cells in the control and ADI (0.1 and 0.2 mL/10 g) groups were arranged neatly, with no bleeding, edoema or other abnormalities. However, there was obvious pathological damage to cardiac tissue from mice treated with DOX. Myocardial cell damage was indicated by the indistinct texture of myocardial tissue, vacuolisation and plasma membrane disruption. High-dose ADI (0.2 mL/10 g) reduced DOX-induced changes in H&E staining and the extent of vacuolar degeneration better than low-dose ADI (0.1 mL/10 g).

**Figure 3.**
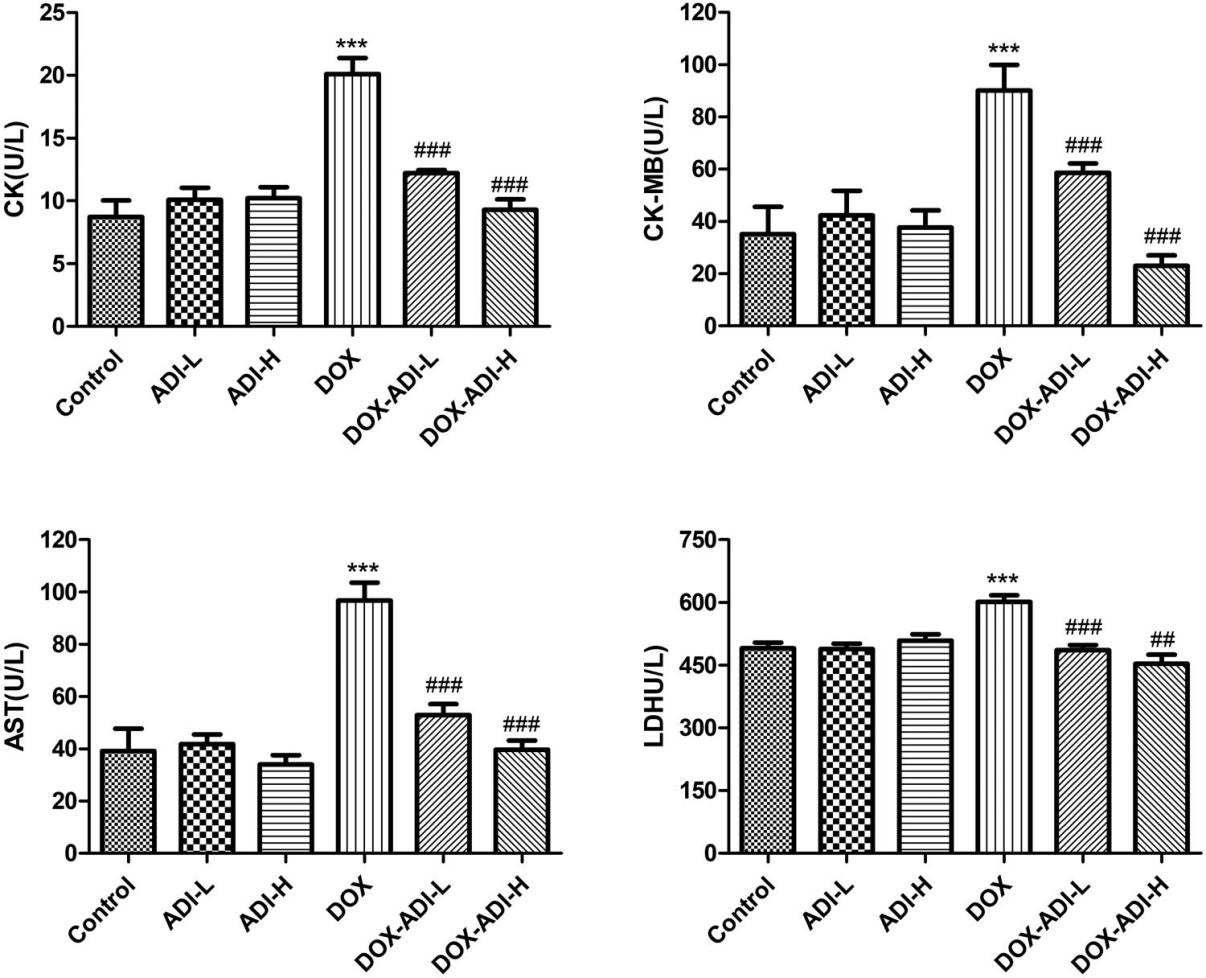
The concentrations of CK, CK-MB, AST and LDH in the serum of mice treated with DOX (0.03 mg/10 g) and/or ADI (0.1 and 0.2 mL/10 g). Data are presented as mean ± SD (*n* = 6). *P < 0.05, ***P < 0.001 vs. control group without DOX treatment; ^#^*P* < 0.05, ^##^*P* < 0.01, ^###^*P* < 0.001 vs. control group with DOX treatment.

As the main biochemical indicators of myocardial damage, serum levels of CK, CK-MB, AST and LDH were examined in the experimental animals and the results are shown in Figure 3. After 2 weeks of treatment, ADI (0.1 and 0.2 mL/10 g/d, *i.p.*) did not affect the plasma levels of CK, CK-MB, AST or LDH. Treatment with DOX (0.03 mg/10 g every other day, *i.p.*) significantly increased the plasma levels of CK, CK-MB, AST and LDH to 2.31, 2.57, 2.47 and 1.23 times that of the control group, respectively. High-dose ADI (0.2 mL/10 g) decreased the DOX-induced up-regulation of these four enzymes better than low-dose ADI (0.1 mL/10 g). In the 0.2 mL/10 g ADI + DOX group, the levels of myocardial enzymes returned almost to their normal levels (Figure 3), indicating that ADI restored cardiac damage.

**Figure 4.**
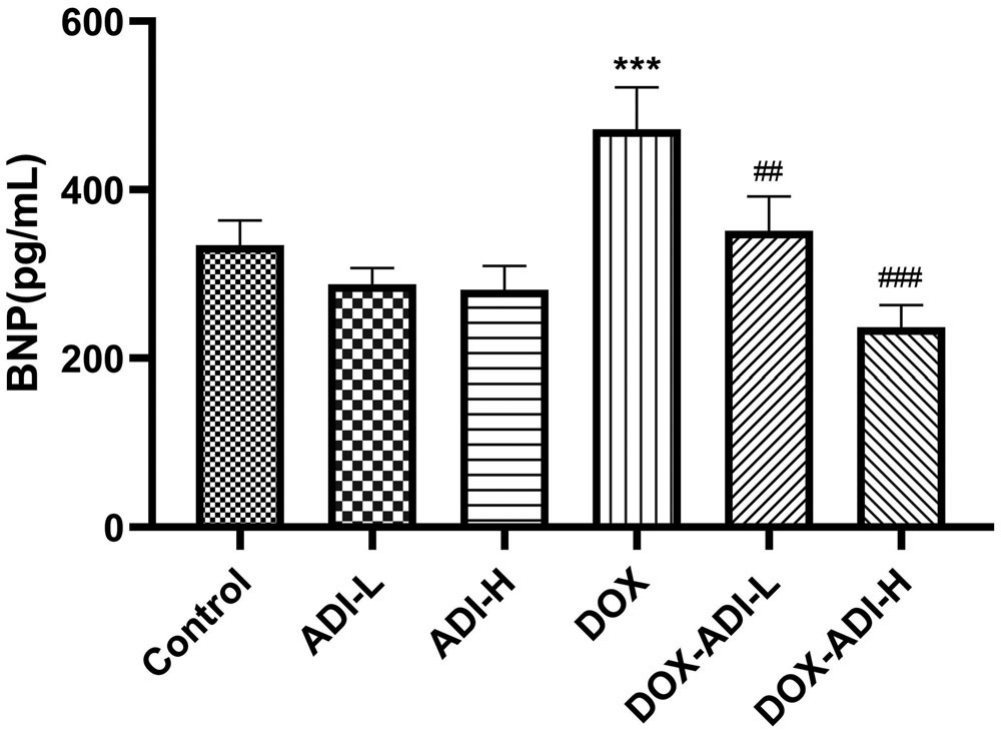
The concentration of BNP in the serum of mice treated with DOX (0.03 mg/10 g) and/or ADI (0.2 mL/10 g). Data are presented as mean ± SD (*n* = 6). ***P < 0.001 vs. control group without DOX treatment; ^##^*P* < 0.01, ^###^*P* < 0.001 vs. control group with DOX treatment.

It is generally believed that the serum level of BNP can be used for diagnosis and as an indicator of the severity and prognosis of heart failure (Kuwahara et al. 2018). In parallel with the myocardial enzyme data, serum BNP levels in the DOX group increased significantly by 1.41 times (Figure 4). Low- and high-dose ADI markedly reduced the serum BNP level by 25.6 and 49.8% compared to DOX group.

### ADI decreased accumulation of the metabolite, DOXol, in heart and serum

**Figure 5.**
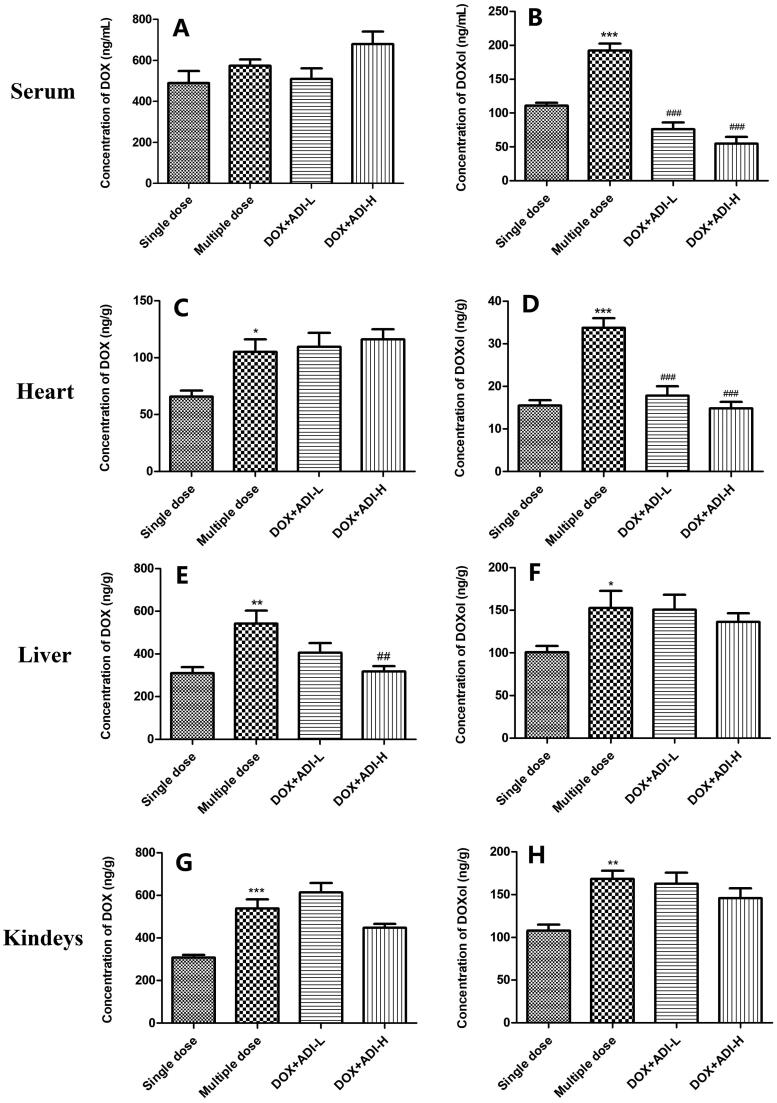
Concentrations of DOX and DOXol in the serum, heart, liver, and kidneys of mice treated with DOX alone or combined with ADI. DOX (A) and DOXol (B) concentrations in mice serum. DOX (C) and DOXol (D) concentrations in heart. DOX (E) and DOXol (F) concentrations in liver. DOX (G) and DOXol (H) concentrations in kidneys. Values are presented as mean ± SD (*n* = 6). *P < 0.05, **P < 0.01, ***P < 0.001 vs. single-dose administration of DOX; ^##^*P* < 0.01, ^###^*P* < 0.001 vs. multiple-dose administration of DOX.

As reported in the literature (Mei et al. 2019) dose-related DOX cardiotoxicity is related to the accumulation of DOX and DOXol in the heart. After repeated administration of DOX, the concentrations of DOX in heart, liver and kidneys were clearly different to those of the single-dose group (Figure 5C, E, G). In addition, compared with single-dose administration of DOX, DOXol concentrations in serum, heart, liver and kidneys were increased by 1.62, 2.48, 1.52 and 1.56 times following multiple-dose administration (Figure 5B, D, F, H). Low doses of ADI did not significantly affect the concentrations of DOX in mouse serum, heart, liver or kidneys when compared with the multiple-dose DOX group (Figure 5A, C, E, G). High doses of ADI did reduce the concentration of DOX in the liver (Figure 5E). However, low- and high-dose ADI markedly reduced the concentration of DOXol in the heart by 47.2 and 59.8% compared to the multiple-dose DOX group (Figure 5D). This may be the mechanism by which ADI reduces DOX-induced cardiotoxicity. Low- and high-dose ADI reduced the concentration of DOXol in the serum by 66.8 and 72.5% compared to the multiple-dose DOX group (Figure 5B), but did not affect DOXol concentrations in the liver or kidneys (Figure 5F and H).

### ADI reduced DOX-induced cytotoxicity in H9c2 cells

**Figure 6.**
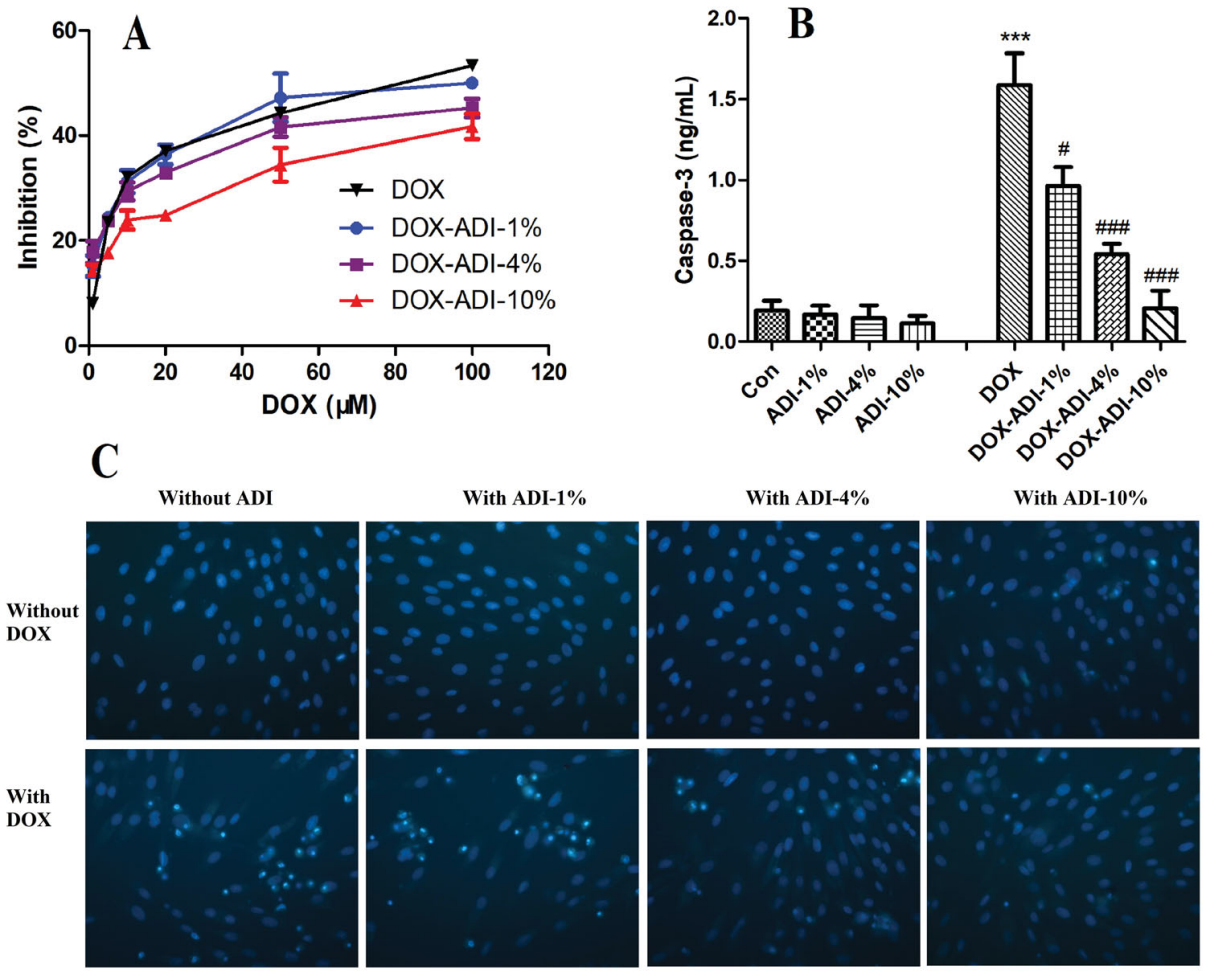
The protective effects of ADI on DOX-induced cytotoxicity in H9c2 cells. (A) Logarithmic plots of the inhibition curves obtained after treating the cells with DOX (1, 5, 10, 20, 50 and 100 μM) in the presence or absence of ADI (1, 4, 10%). (B) Effect of ADI on caspase-3 activity induced by DOX (5 μM) in H9c2 cells. (C) Representative fluorescence images of Hoechst 33342 staining of H9c2 cells after different treatments. Magnification: 200× Values are expressed as mean ± SD from six independent experiments. **P < 0.01, ***P < 0.001 vs. control group without DOX, ^###^*P* < 0.001 vs. control group with DOX.

The results from the MTS assay showed that DOX (1, 5, 10, 20, 50 and 100 μM) concentration-dependently reduced the survival of H9c2 cells (Figure 6A). Combined treatment with ADI (1, 4 and 10%) improved cell viability in a concentration-dependent manner. The IC_50_ values of DOX in the DOX, DOX-ADI-1%, DOX-ADI-4% and DOX-ADI-10% groups were 68.51, 83.47, 176.9 and 310.8 μM, respectively.

As shown in Figure 6B, DOX (5 μM) significantly increased the activity of caspase-3 in H9c2 cells by 8.23 times (*P* < 0.001), while ADI (1, 4 and 10%) alone had no significant effect. When DOX was combined with ADI (1, 4 and 10%), the caspase-3 activity decreased by 39.17, 43.96 and 61.82%, respectively.

Hoechst 33342 staining was also performed to investigate morphological changes of nuclei subjected to DOX-induced apoptosis (Figure 6C). The nuclei of the control group were uniformly blue, oval, and well-defined, indicating that the cells were growing well. There were no obvious changes when the cells were treated with ADI alone (1, 4, 10%). After DOX (5 μM) treatment, however, many cells exhibited pyknosis and nuclear fragmentation. Combined treatment with ADI (1, 4, 10%) alleviated the DOX-induced morphological changes. The results indicated that ADI specifically reduced DOX-induced cardiotoxicity.

### ADI altered the concentration of DOXol in H9c2 cells

**Figure 7.**
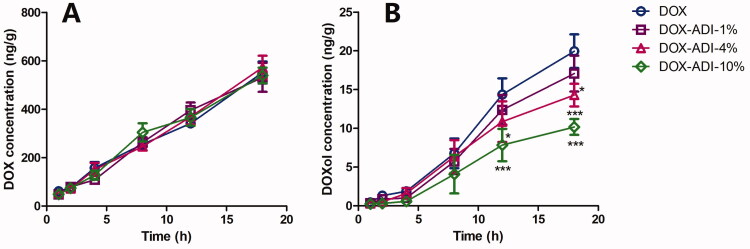
Time-concentration curves of DOX (A) and DOXol (B) in H9c2 cells. H9c2 cells were incubated with 5 μM DOX in the absence or presence of 1, 4, or 10% ADI for 1, 2, 4, 8, 12 and 18 h. DOX and DOXol concentrations in cells were quantified by UPLC-MS/MS. Data are expressed as mean ± SD of six independent experiments. **P < 0.01, ***P < 0.001 vs. DOX group at the same time.

H9c2 cells were incubated with DOX (5 μM) in the absence or presence of 1, 4, or 10% ADI for 1, 2, 4, 8, 12 and 18 h. DOX and DOXol concentrations in cells were quantified by UPLC-MS/MS. As shown in Figure 7, ADI (1, 4, 10%) did not affect the accumulation of DOX in H9c2 cells. However, ADI (1, 4, 10%) treatment decreased the cumulative amount of DOXol in H9c2 cells by 14.45, 28.39 and 48.99%, respectively.

### ADI inhibited the expression of CBR1 protein in H9c2 cells

**Figure 8.**
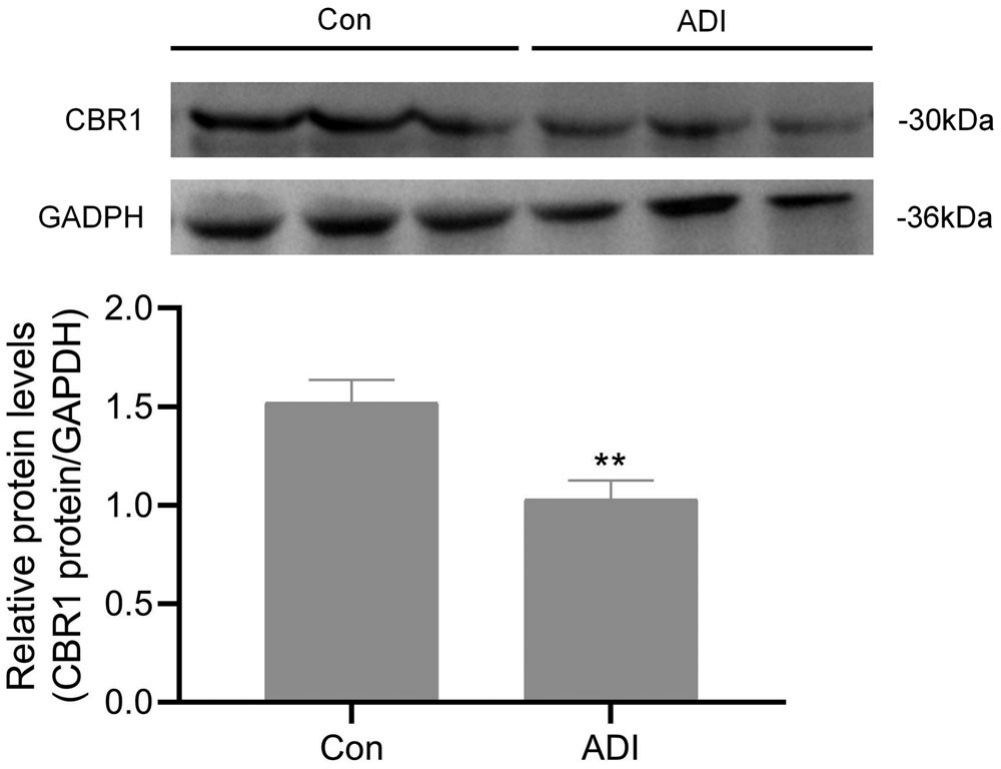
Effects of ADI on the expression of CBR1 in H9c2 cells. H9c2 cells were incubated with ADI (10%) or an equivalent volume of PBS for 18 h. CBR1 protein levels was determined by western blot analysis. (A) Representive images of CBR1 expression detected by Western blotting. (B) Protein ratios normalised to GAPDH used to quantify fold change by densitometry and shown in a histogram. Data are expressed as mean ± SD of three independent experiments. **P < 0.01 vs. control group at the same time.

CBR1 contributes mainly to the conversion of DOX to DOXol (Lal et al. 2010). To determine whether ADI inhibit the expression of CBR1 protein, we performed Western blot analyses using H9c2 cells. As shown in Figure 8, compared with the control group, ADI (10%) inhibited the expression of CBR1 protein in H9c2 cells by 32.3%.

## Discussion

Malignant tumours present a serious health threat and have now become the number one cause of death in humans. Anthracyclines are some of the most effective chemotherapeutics for the treatment of malignant tumours, and are widely used clinically to treat hematological malignancies and solid tumours (Martins-Teixeira and Carvalho 2020). However, anthracyclines are potentially cardiotoxic. As early as 1966, studies reported that anthracycline treatment could induce myocardial injury. Cardiotoxicity has been observed in up to 30% of patients, and the changes were irreversible. At present, the dose-dependent irreversible myocardial injury caused by anthracyclines is an important factor limiting their clinical application. There is no effective treatment for anthracycline-induced cardiotoxicity, so early monitoring and prevention are particularly important. DOX is a representative anthracycline and its metabolism has been thoroughly studied (Rawat et al. 2021). DOX is mainly metabolised by carbonyl reductase 1 (CBR1) and is excreted in the bile. Approximately 50% is excreted as the parent drug and 23% as the active metabolite, DOXol (Lal et al. 2010). Many studies have shown that DOXol has lower cytotoxicity in tumour tissues than the parent drug DOX, but its myocardial toxicity is much higher than that of DOX (Olson et al. 1988; Minotti et al. 2001). Both DOX and DOXol can damage mitochondria, reduce the production of ATP and lead to the formation of reactive oxygen species (ROS) and oxidative stress. They have also been shown to induce apoptosis and dysregulate cells by regulating ion channels, membrane transporters and calcium pumps, disrupting calcium homeostasis (including in mitochondria; Wallace et al. 2020).

In recent years, advances in theoretical research on Chinese medicine have led to significant progress in the treatment of malignant tumours, and Chinese medicine has become an important part of their treatment in China (Xiang et al. 2019). Traditional Chinese medicine injections have advantages that include a high effective concentration, rapid effect, and convenient administration for patients with advanced tumours who cannot swallow, so they are widely used clinically (Qi et al. 2015). ADI is widely used in China as an early antitumor treatment. At present, most reports concerning ADI are observations of clinical efficacy and retrospective literature analysis. For example, ADI combined with CHOP chemotherapy (doxorubicin-based chemotherapy protocols containing cyclophosphamide, vincristine and prednisone) improved the treatment of malignant lymphoma and patient quality of life, and reduced the risk of severe leukopoenia or thrombocytopenia (Lu et al. 2016; Wang et al. 2016). ADI combined with TACE (transarterial chemoembolization) significantly improved therapeutic efficacy in liver cancer and was better than TACE alone (Chen et al. 2018), improved the overall survival rate (Dai et al. 2018) and reduced the incidence of adverse events (Shen et al. 2017). Compared with platinum alone, combination of ADI with platinum chemotherapy improved clinical efficacy in patients with stage IIIB/IV non-small cell lung cancer and reduced the toxicity of chemotherapy (Wang et al. 2018). These studies demonstrate that ADI has a wide range of clinical anti-tumour applications and definite therapeutic effects. By reducing the side effects of chemotherapy, it can improve patient quality of life. However, the quality of the literature is not very high and, in particular, there is insufficient evidence that ADI is able to reduce the side effects of chemotherapy drugs. In addition, there is currently no basic research showing that ADI can reduce the myocardial toxicity of DOX.

Therefore, in the present study, we have used a mouse model of DOX-induced myocardial injury to investigate the effects and mechanism of ADI. The dosage of DOX was screened in a preliminary experiment using 2, 3 and 4 mg/kg. The 2 mg/kg dose was too low, with no obvious myocardial toxicity observed. About 30% of the mice were intolerant to 4 mg/kg DOX and died during the experiment. Consequently, a dose of 3 mg/kg was selected for the myocardial injury model. The results from scanning electron microscopy after 15 days of repeated DOX administration showed that the cardiomyocytes of the mice were damaged. The mitochondrial morphology in the model group was abnormal, with the mitochondria appearing swollen. The serum levels of CK, CK-MB, AST and LDH in the model group increased significantly, indicating that the cardiomyocytes were seriously damaged. The levels of BNP, a well-recognized marker of heart failure (Kuwahara et al. 2018), in the model mice were significantly increased, suggesting that the mice may have signs of heart failure. We did not, however, use echocardiography to confirm whether heart failure had occurred. Nevertheless, the above points indicate that the DOX-induced myocardial injury model was successfully established and could be used for subsequent evaluation of ADI. The tissue distribution experiments showed that compared with a single dose of DOX, DOX and DOXol levels in the heart, kidney and liver were significantly increased after multiple doses of DOX, confirming the dose-dependent cardiotoxic effects of DOX.

In the clinical treatment of hepatocellular carcinoma, ADI is dosed simultaneously with chemotherapy drugs for a period of 10–30 days. Therefore, the mice were dosed with ADI for 15 consecutive days in this experiment. After 15 days of ADI treatment, H&E staining showed that ADI reduced DOX-induced myocardial damage. After treatment with ADI, the serum levels of CK, CK-MB, AST and LDH in the model animals were decreased. ADI also reduced the serum level of BNP in model mice. The results showed that ADI effectively alleviated the myocardial toxicity caused by DOX in animals. In cell experiments, compared with DOX alone, the combined use of ADI and DOX increased the survival of H9c2 cells, reduced caspase-3 activity and improved nuclear morphology in a dose-dependent manner. In summary, the results from the animal model and cell experiments showed that ADI can reduce the myocardial toxicity of the chemotherapy drug, DOX.

It is generally believed that the pharmacological effects of drugs are positively correlated with the drug concentration in plasma or target tissues, and also positively correlated with the intracellular drug concentration. We therefore used UPLC-MS/MS to determine the drug concentrations in tissues, organs and myocardial cells. The method used to determine DOX and DOXol in plasma and tissues was an established method (Lu et al. 2018). To measure the concentrations of DOX and DOXol in H9c2 cells, the cells must first be lysed. Commonly used methods for cell lysis include repeated freeze-thawing, ultrasonic cell lysis and the use of lysis solution. Since inorganic salts should be avoided as much as possible for UPLC-MS/MS, the lysis solution method was not chosen. Preliminary experiments compared treatment of cells with repeated freeze-thawing and ultrasonic disintegration. Since there were no significant differences between the methods according to UPLC-MS/MS analysis, we chose the simple repeated freeze-thawing method that could be operated in batches. The tissue distribution results showed that high-dose ADI reduced concentrations of DOX in the liver, but had no significant effects on DOX concentrations in serum, heart or kidney. Both high and low doses of ADI reduced the concentrations of DOXol in serum and heart but had no significant effects on the concentrations of DOXol in liver or kidney. Cell studies showed that, at the end of the experiment, the concentrations of DOX were not significantly different in cells from the low, medium, and high ADI combined with DOX dose groups compared with DOX alone. The concentration of DOXol in the cells, however, was dose-dependently reduced. The animal experiment and cell data indicated that ADI did not affect the total amount of DOX in myocardial tissues and cells, but reduced the accumulation of the toxic metabolite, DOXol, that may be the most important cause of oxidative stress damage. DOX is mainly metabolised by CBR1 and CBR3 to produce DOXol (Lal et al. 2010), so we speculated that ADI might reduce the accumulation of DOXol in heart tissues and cardiomyocytes by changing the expression or activity of CBR1 or CBR3, thereby reducing oxidative stress and, ultimately, toxic effects. It was confirmed in western blot analysis that ADI inhibited the expression of CBR1 protein in H9c2 cells.

Various components in *P. ginseng* and *A. membranaceus* have been reported to reduce DOX-induced myocardial toxicity. For example, ginsenoside Rg1 prevents DOX-induced cardiotoxicity by inhibiting autophagy and endoplasmic reticulum stress in mice (Xu et al. 2018); ginsenoside Rb1 has a protective effect on DOX-induced cardiac autophagy (Li et al. 2017); ginsenoside Rg3 regulates the Nrf2-ARE pathway by up-regulating Nkt and activating Akt, thereby improving endothelial dysfunction caused by oxidative stress and antagonising DOX-induced cardiotoxicity (Wang et al. 2015). *Astragalus* polysaccharides restore autophagy flux and improve cardiomyocyte function in DOX-induced cardiotoxicity (Cao et al. 2017). *Astragalus* polysaccharide inhibits DOX-induced cardiotoxicity by regulating PI3k/Akt and p38MAPK pathways (Cao et al. 2014). Astragaloside IV inhibits DOX-induced cardiomyocyte apoptosis by activating the PI3K/Akt pathway (Jia et al. 2014).

## Conclusions

ADI reduced DOXol concentrations in myocardial tissue and cardiomyocytes by inhibiting CBR1 protein expression, thereby reducing DOX-induced cardiotoxicity. This research provides a scientific basis for the clinical use of ADI and DOX.
